# Emotional and behavioural symptoms in childhood (aged 8–14) and severity of mental illness outcomes in young adults: a retrospective observational study using cross-sectional data from electronic health records in the clinical practice research datalink (CPRD)

**DOI:** 10.1186/s12887-025-06425-7

**Published:** 2026-01-16

**Authors:** Ana Pascual-Sanchez, Geva Greenfield, Hanna-Marie Creese, Sonia Saxena, Dougal Hargreaves, Dasha Nicholls

**Affiliations:** 1 Department of Brain Sciences, Division of Psychiatry, Imperial College London, 2nd Floor Commonwealth Building, Du Cane Road, London, W12 0NN United Kingdom; 2 CAMHS DBT Service, East London NHS Foundation Trust, London, United Kingdom; 3 Department of Primary Care and Public Health, Imperial College London, London, United Kingdom; 4 Department of Epidemiology and Biostatistics, School of Public Health, Imperial College London, London, United Kingdom

**Keywords:** Primary care, Severe mental illness, Adolescence, Emotional dysregulation, Self-harm

## Abstract

**Background:**

This study aimed to investigate the prevalence and impact of emotional and behavioural dysregulation (EBD) symptoms in childhood/early adolescence reported in primary care among young adults with severe mental illness.

**Methods:**

Data were from electronic health records from general practices registered on the Clinical Practice Research datalink (CPRD) from 1987 to 2016 on young adults (aged 18 to 24 years) with mental illness who also had at least one primary care consultation for any reason while they were 8 to 14 years old. Using logistic regression, we examined the association of several EBD symptoms at age 8–14 years with severe mental illness rather than common mental illness in early adulthood.

**Results:**

161,654 young adults aged 18–24 with mental disorder were included in the analysis (63.9% females; 9.4% classified severe). 19.0% had consultations for EBD symptoms at age 8–14. EBD symptoms in childhood/early adolescence increased the likelihood of later severe rather than common mental illness (OR = 1.30, CI: 1.20–1.41). Among symptoms which predicted a higher likelihood of severe mental illness were self-harm (OR = 3.05, CI: 2.06–4.73), suicidal ideation (OR = 2.34 CI: 1.15–4.76), behavioural problems (1.43, CI: 1.22–1.67) and mood symptoms (OR = 1.39, CI: 1.08–1.80). Anxiety, sleep difficulties, eating symptoms and hyperactive behaviours in childhood were not significantly associated with severe mental illness in young adults.

**Conclusion:**

Primary care consultations for EBD symptoms, particularly self-harm, suicidal ideation, behavioural problems and mood symptoms, at age 8–14 years are associated with poorer mental health in young adulthood.

**Supplementary Information:**

The online version contains supplementary material available at 10.1186/s12887-025-06425-7.

## Introduction

Evidence on early detection and intervention of mental health problems and their associated functional impairment to reduce/prevent the growing mental health burden in adolescents and young adults, and the subsequent social and financial impact, is urgently needed. Three quarters of all mental health problems start before the age of 25 years and 50% before the mid-teens [1], making late childhood and early adolescence a key period for identification and prevention efforts for those at risk. There has been an increase in emotional disorders in childhood, from 3.9% in 2004 to 6.0% in 2017 [2]. Emotional and behavioural problems are among the most common reasons for children to seek help from health services, including primary care, but little is known about whether these presentations are early signals for those children who go on to develop severe mental illness. In the UK in 2023, one in five children and young people aged 8 to 25 presented with enough symptoms and impairment to likely meet criteria for a mental disorder [3].

Raballo, Schultze-Lutter & Armando [4] suggested that the current direction of research addressing early detection of severe mental illness has started to encourage an approach based on subtle psychopathological antecedents (e.g., before they reach the point of higher illness severity) to identify young people at higher risk of severe mental illness. This is highly relevant since despite the lack of a consensus on a definition that clearly differentiates common and severe mental illness, particularly when the diagnoses that have been traditionally considered common mental illness (depressive and anxiety disorders) can also range in severity, the evidence suggests that severe mental illness is often associated with pervasive difficulties and significant impairment in daily functioning. It has also been commonly named as severe and persistent mental illness [5] and the use of diagnoses associated with this chronic prognosis (e.g., psychotic disorders, bipolar disorders, personality disorders, eating disorders) as part of this category for research studies and when commissioning specialist services. Thus, better profiling of children and adolescents who present to primary care with emotional and behavioural problems who may be at risk of severe mental illness might enable detection and early intervention for some children, reducing the risk of chronicity and impact on functioning in later life. In addition, healthcare costs are significantly higher for those young people with higher illness severity, since they are more likely to require specialist care in adult mental health services than those with less severity of symptoms in childhood and adolescence [6].

Emotion dysregulation is defined as pattern of difficulties to recognize, evaluate and manage emotions that interfere with goal-directed activity, which occurs in the context of the interaction of the biological vulnerability and the environment the individual lives in [7]. This was originally described by Linehan [8] in the biosocial model of emotional dysregulation, and provides the foundation for early detection of symptoms that could suggest vulnerability to further develop into pervasive severe mental illness later in life. Emotional and behavioural dysregulation (EBD) can be considered an umbrella term that can include several areas of dysregulation (e.g., behavioural dysregulation, cognitive dysregulation), with emotional dysregulation at the core. This has been the focus of targeted interventions such as Dialectical Behavioural Therapy (DBT), initially developed for adults with borderline personality disorder [8] and then adapted to other populations with similar core symptoms, including adolescents with pervasive emotional dysregulation [9] where certain diagnosis (e.g., personality disorder) are not given due to the lack of longitudinal stability of those difficulties during adolescence, that may either become chronic later in life or improve significantly with treatment [10]. EBD underlies multiple types of psychopathology, such as anxiety, mood disorders, eating disorders, self-harm or risk of suicide [11, 12]. Emotional dysregulation has been increasingly considered a transdiagnostic trait in young people’s mental health and has been associated with higher severity of impairment as well as longer treatment duration [13]. EBD is common in severe mental illnesses, including schizophrenia, bipolar disorder, and borderline personality disorder [14]. EBD symptoms are also potentially important early markers of mental disorder and have become recognised as important targets for prevention strategies [15].

Furthermore, somatic symptoms (e.g. headache, abdominal pain) in adolescence, measured at school, predicted severe mental illness in young adulthood [16]. A hypothesis is that functional somatic symptoms, which often start in childhood and adolescence, may be associated with limited emotional regulation and certain responses from the environment; those factors seem to play a relevant role in the development and maintenance of these somatic symptoms [17].

However, recent studies in the UK using primary care data suggest that symptoms in childhood that require GP appointments for EBS symptoms do often not require further GP input 5 years after, although it is unclear if the needs were met elsewhere or what were the long-term mental health outcomes on this population in early adulthood, e.g., requiring specialist input [18]. On the contrary, other studies have identified that increased risk to self (e.g., self-harm, suicidal ideation), others (behavioural problems) or from others (e.g., sexual abuse) between the age of 8–14 predicted later self-harming behaviours in young adults [19]. However, the predictive value of primary care consultations for those symptoms in late childhood and early adolescence to identify potential risk of mental illness severity at the age of 18–24 has not been studied. It is not uncommon for parents to seek help for their children from their GP for emotional or behavioural difficulties, or for emotional factors that may play a role in presentations for physical complaints [20]. Analysing how common these symptoms are as presentations in primary care for children and adolescents, and how these presentations relate to later mental health outcomes, including mental illness severity, has been relatively little studied.

To our knowledge no previous study has examined if certain symptoms that motivate primary care presentations in late childhood and early adolescence (8–14 years old) may be early signs of later severe mental illnesses. This exploratory study, based on retrospective primary care records, aimed to: (1) describe the prevalence of different types of earlier EBD symptoms in young adults with mental illness; (2) explore links between consultations for EBD symptoms in childhood and adolescence and risk of severity mental illness diagnoses in young adulthood. We hypothesised a relationship for EBD symptoms with severe mental illnesses particularly for those that involved higher risk to self (e.g., self-harm, suicidal ideation) and others (e.g., behavioural problems).

## Methods

### Study design

**Fig. 1:**
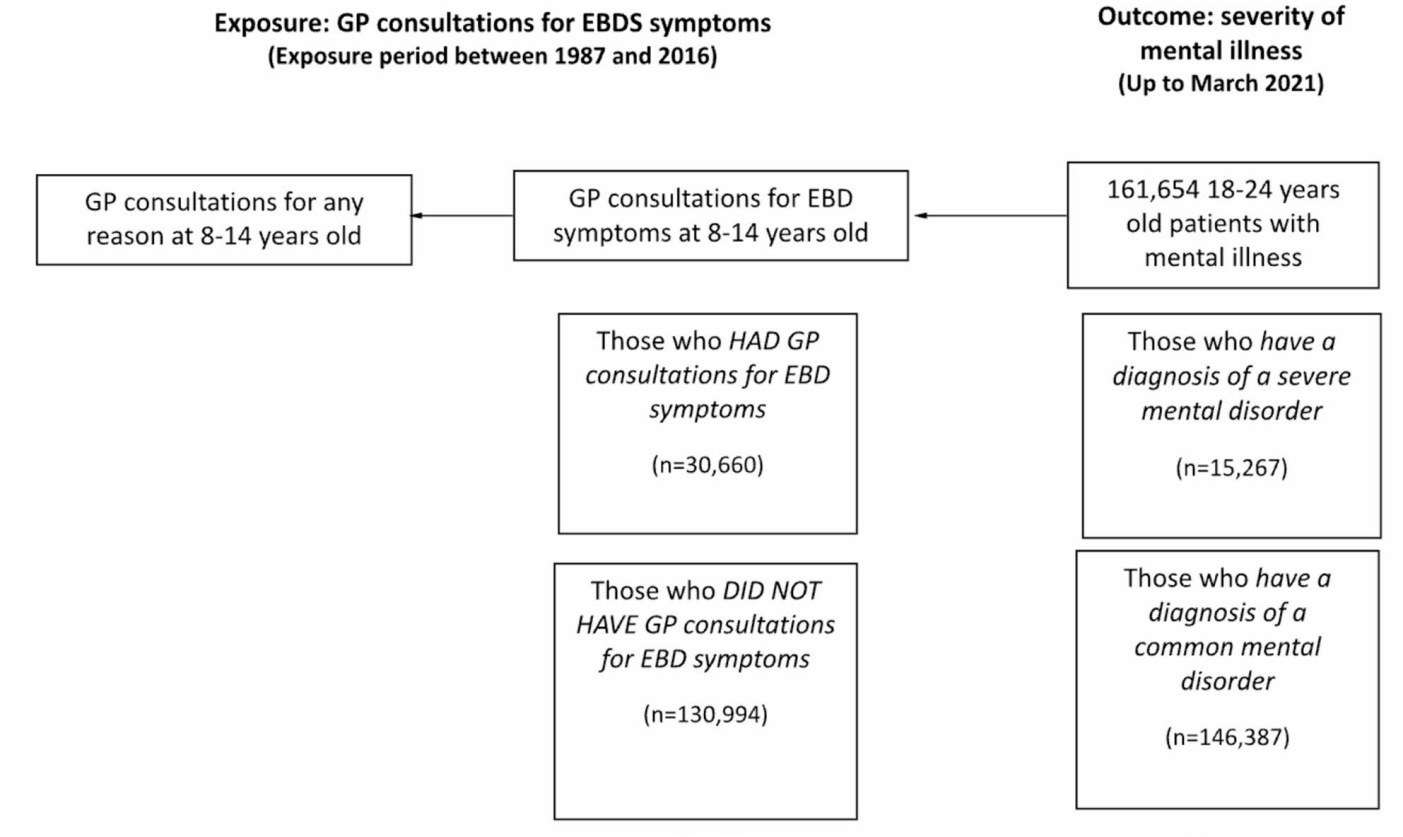
Retrospective observational study design: exposure and outcomes description

We conducted a population-based retrospective observational study of young adults with mental illness (see Fig. 1) using longitudinal, patient-level data from the Clinical Practice Research Datalink (CPRD). CPRD includes de-identified data on patient demographics, primary care consultations and secondary care referrals. CPRD is the largest validated primary care research database in the UK, representative for age, gender and ethnicity and representing 8% of the UK population [21]. The study period was January 1987 (when CPRD collection started) to March 2021. In order to include only those who have data in the exposure period (Fig. 1), we included only those participants who had GP health records from when they were aged between 8 and 14 years. to capture the developmental window of late childhood through early adolescence, a critical period for the emergence of emotional and behavioural difficulties that often precede diagnosable mental disorders in early adulthood (18 to 24), where most mental illnesses develop. Prior to age eight, the identification of internalizing symptoms (e.g., anxiety, low mood) is limited by children’s restricted capacity for accurate self-report and the heavy reliance on parental observation, which may under-detect early emotional distress [22]. The upper boundary of 14 years was selected because it marks the transition between early adolescence to mid and later adolescence, during which many individuals already develop clinically recognizable disorders [23]. Focusing on the 8–14 age window therefore aligns with the study’s aim of early identification of risk. Tracking GP consultations for EBS symptoms during this period allows identification of individuals exhibiting early help-seeking behaviour or subclinical manifestations of distress that may signal vulnerability to later severe mental illness.

### Participants, exposures, outcomes and covariates

#### Participants

We included participants that were registered with CPRD partnered GP practices and have data in CPRD GOLD who had consultations between age 18 and 24 for a diagnosed mental disorder, subcategorised as common (e.g. depression, anxiety) or severe (e.g. psychosis, personality disorder, eating disorder).

#### Exposures

Within the included sample, we compared those who had EBD symptoms at age 8–14 with those who did not have consultations for those reasons, based on Read codes for consultations for potential EBD symptoms (Appendix A.1.). Read codes are a coded thesaurus of clinical terms to record patient findings in healthcare IT systems in the NHS. Candidate markers as proxy EBD symptoms were identified from previous literature [24]. Final codes were then selected by an expert group of practising child and adolescent mental health clinicians based on clinical experience and research literature. Initially the codes were selected by introducing in the code browser the key symptoms that are representative of EBD symptoms and selecting those occurring in multiple consultations (Appendix A.1). Categories of EBD symptoms with relevant Read codes include somatic symptoms, anxiety symptoms, mood symptoms, eating symptoms, sleep symptoms, behavioural symptoms, hyperactivity symptoms, self-harm and suicidal ideation. Some of these symptoms (e.g., somatic symptoms) could also signify physical health diagnoses, which in turn increase the risk of mental illness. This risk was partially mitigated by controlling for common chronic physical health comorbidities (Appendix A.3.).

#### Severity outcomes

Within the sample with proxy codes for mental illness at age 18–24 years, we defined severity as *specified mental disorder diagnosis* Read codes (Appendix A.2.). We created a binary variable for severe mental illness (including psychotic disorders, personality disorders and eating disorders) versus common mental illness codes.

#### Covariates

In order to reduce the likelihood that somatic symptoms were markers of physical rather than mental disorders, we adjusted for comorbid conditions (Appendix A.3.) diagnosed before the age of 25 including neurodevelopmental disorders, neurological disorders (e.g. cerebral palsy) and long-term physical illness, based on Hardelid classification [25] that might account for increased EBD symptoms, particularly somatic symptoms. We also identified gender, socioeconomic status (using patient Level Index of Multiple Deprivation (IMD) and practice level for those where the patient level data was missed) and ethnicity (see descriptives for categorisation details) as potential confounders.

### Statistical analysis

#### Descriptive analyses

To estimate the prevalence of primary care consultations for EBD symptoms in childhood and adolescence among those who have mental illness in young adulthood, we calculated the total consultations for these reasons at the age of 8–14 years.

#### Regression analyses

To identify if earlier EBD symptoms were linked to later mental illness, we calculated the odd ratios for having severe mental illness (vs. common mental illness) by comparing those with primary care consultations for specific types of EBD symptoms in late childhood/early adolescence (age 8–14 years) with those who did not have those symptoms (Fig. 1). We used Generalized Estimating Equations (GEE) for logistic regression to account for practice ID. We adjusted for gender, age of first GP consultation within the study period, comorbidity, IMD and ethnicity. 

## Results

We identified 161,654 participants aged between 18 and 24 years as having a mental illness who were eligible for inclusion in the study from CPRD GOLD. Of these, 63.6% were females and 8.7% had a comorbidity diagnosed before the age of 25 years (Table 1).

**Table 1:** Sample characteristics: young adults (age 18–24) with mental illness (*n* = 161,654)

		*n*	%
Gender	Female	102,789	36.41
	Male	58,852	63.59
	Indeterminate	13	0.01
Comorbidity		14,125	8.74
Index of Multiple Deprivation (IMD) *^a^(patient or practice level data) (*n* = 74,700)	1	11,367	15.22
	2	13,998	18.74
	3	14,231	19.05
	4	15,249	20.41
	5	19,855	26.58
Ethnicity* (*n* = 47,790)	White	34,193	93.10
	Black	397	1.08
	Asian	462	1.00
	Other	290	0.79
	Mixed	505	1.38
	Unknown	879	2.39
Number of patients with a severe mental disorder diagnosis		15,267	9.44
Number of patients with at least one visit for an early marker of EBD at age 8–14		30,660	18.97
Number of patients with EBD with somatic symptoms at the age of 8–14	Somatic symptoms indicative of possible EBD	13,256	43.24
	Non-somatic EBD symptoms	17,404	56.76
Number of visits for early marker of EBDS at the age of 8–14	1	20,458	66.73
	2	5,774	18.83
	3	2,110	6.88
	4	1,025	3.34
	5	525	1.71
	≥ 6	768	2.11

*Ethnicity and IMD were not available in CPRD GOLD (just linked data) – *n* = 74,700 with IMDdata ( *n* = 36,726 with ethnicity data)

^a^Index of Multiple Deprivation

### Prevalence of GP consultations for EBD symptoms at the age of 8–14

19% of included participants had at least one visit for proxy EBD symptoms at the age of 8–14 (Table 1). 66.7% of patients with consultations for EBD symptoms had just one visit for this reason, 18.8% two visits and 14.5% three visits or more. 

Of the visits for EBD symptoms as defined, 56.76% were for non-somatic symptoms. Of those, the most common symptoms identified in childhood and adolescence were behavioural symptoms, mood symptoms and self-harm (Table 2). A summary of the prevalence of EBD symptoms at age 8–14 for different mental illness severity outcomes at the age of 18–24 can be found in Table 2.

**Table 2:** Description and odd ratios of severity of mental illness at the age of 18–24 depending on the presence of specific emotional and behavioural dysregulation symptoms (EBDS) at the age of 8–14*

VARIABLE	Common mental illness (*n* = 146,387)		Severe mental illness (*n* = 15,267)		OR (95% CI)	*p*
	*n*	%	*n*	%		
Anxiety symptoms (*n* = 1,066)	913	0.62	153	1.00	1.01 (0.69–1.47)	0.001
Mood symptoms (*n* = 1,875)	1,554	1.06	321	2.10	1.40 (1.08–1.80)	< 0.001
Eating symptoms (*n* = 196)	180	0.12	16	0.10	1.28 (0.60–2.74)	0.180
Sleep symptoms (*n* = 224)	200	0.14	24	0.16	1.00 (0.47)	0.829
Behavioural symptoms (*n* = 8,961)	7,851	5.36	1,110	7.27	1.43 (1.22–1.67)	< 0.001
Hyperactivity symptoms (*n*= 165)	151	0.10	14	0.09	0.91 (0.28–2.99)	0.273
Self-harm (*n* = 769)	570	0.39	199	1.3	3.05 (2.06–4.54)	< 0.001
Suicidal ideation (*n* = 215)	163	0.11	52	0.35	2.34 (1.15–4.77)	< 0.001
Somatic symptoms	11,418	7.80	1,839	12.05	1.62 (1.54–1.69)	0.001

*Covariates: gender, age of first GP consultation within the study period, chronic comorbiditydiagnosed before the age of 25, IMD and ethnicity

### Early predictors of severe mental illness and risk behaviours amongst young adults with mental illness

Between age 18 and 24, 9.44% of the sample had a severe mental disorder diagnosis recorded at a primary care visit, the remaining 90.56% having common mental disorders (Appendix A.2.). Half of the sample (50.35%) with a diagnosed mental disorder attended primary care for the first time in early adulthood between the ages of 18 and 20 (out of the 18–24 cohort) (Appendix B.2.).

Logistic regression analyses showed that having at least one consultation for one EBD symptom or more between 8 and 14 years increased the likelihood of later severe, rather than common, mental illness (OR = 1.30, CI: 1.20–1.41).

When we analysed the association between specific categories of symptoms in late childhood and early adolescence with severe mental illness, we found that consultations for somatic symptoms (headache, abdominal pain) were more likely to be present between the ages of 8–14 in those with a later diagnosis of severe mental illness than in those with common mental illness (OR = 1.62, 1.54–1.69). Symptoms associated with increased risk to self, such as self-harm and suicidal ideation, significantly increased the risk of severe mental illness. However, eating, sleep and hyperactivity symptoms did not appear to predict increased likelihood of severe mental illness (OR 0.91–1.28) when present at the age of 8–14 (see Table 2).

## Discussion

### Main findings

To our knowledge, this is the first study using a large and representative sample of routine healthcare data exploring the relationship between primary care consultations for EBD symptoms in late childhood and early adolescence and the likelihood of severe mental illness in young adulthood across decades with a large sample size. We found that those symptoms were identifiable in primary care records, and that some of them were associated with increased risk for later severe mental illness. 

#### Prevalence of EBD symptoms on primary care records

Our findings showed that 19.4% of young adults with later mental illness had at least one consultation in primary care for EBD symptoms in late childhood or early adolescence. This is comparable to previous studies suggesting that 5% of children and adolescents would have primary care records for emotional symptoms [26], whereas this study addresses those with persisting mental difficulties in early adulthood, suggesting a higher proportion of earlier symptoms than the average population. In our study, the most common symptoms were behavioural problems, followed by mood symptoms and self-harm. This provides more nuanced information to detect subtle difficulties and enhances primary care clinicians’ understanding on how EBD symptoms can present in GP consultations.

#### Links between EBD symptoms in childhood and adolescence and mental illness severity in young adulthood

Our findings suggest that primary care consultations for EBD symptoms in late childhood and early adolescence could be potential early markers of risk for later mental illness. This is congruent with previous studies in mental health and school settings that have identified early mental health or somatic symptoms as predictors of later mental health outcomes [16, 27, 28]. A previous study using CPRD found that those with severe mental illness had progressively more attendances in primary care than those without severe mental illness [29]. However, the analyses were not specific to young people (mean age was around 50 years old), nor was the implication of EBD related presentations in childhood and adolescence explored.

Vergunst et al. [30] suggested that children aged 6 to 12 at school who presented high externalising symptoms (e.g., behavioural problems) and high internalising symptoms (e.g., mood symptoms), and particularly more those with both, had poorer long-term economic and social outcomes across early adulthood compared to children without. Furthermore, the association between externalising symptoms and self-harm has been consistently reported in the literature [31]. These findings are consistent with our results of self-harming, behavioural and emotional symptoms being more common in those with severe mental illness.

In our sample, self-harm and suicidal ideation seem to be potential better predictors of later severe mental illness than other symptoms. It was 3 times more common to have severe mental illness in early adulthood in the presence of self-harm in childhood and early adolescence in our sample, and over twice as common if suicidal ideation was present at a similar age. Self-harm and suicidal ideation earlier in life have also been associated with higher risk of self-harming behaviours in early adulthood [19]. Our results are consistent with previous literature that has identified a significant association between emotional dysregulation and self-harm [32], as well as the high prevalence of EBD in severe mental illnesses, including schizophrenia, bipolar disorder, and borderline personality disorder [14]. It is worth considering whether earlier EBD symptoms may be a risk factor that, left untreated, developed into more complex forms of mental illness that required higher intensity of treatment and are associated with higher levels of needs. Despite most interventions for EBD in children and adolescent mental health services being targeted to adolescents, there is a growing field of targeted interventions for emotional dysregulation in older children and early adolescents, such as DBT-C, which have shown promising results in treatment satisfaction and emotional and behavioural symptoms in 7–12 year olds [17]. Furthermore, somatic symptoms, such as headaches or abdominal pain, in childhood and adolescence also increased the likelihood of later mental illness, in line with previous studies [33]. Other studies have also found that somatic symptoms measured at school in late adolescence were associated with an increased risk of later psychiatric inpatient admission in young adults [16]. However, other symptoms commonly associated with mental illness, such as problems with eating, sleep and hyperactivity symptoms were not associated with a higher likelihood of severe mental illness in our sample. It may be that those symptoms were found across all types of mental illness, common and severe, or only associated when the symptoms were severe, which we were unable to quantify due to the nature of the data (e.g., severe restrictive eating, severe sleep deprivation, significant levels of hyperactivity and impulsivity).

In summary, our data show that EBD symptoms in late childhood and early adolescence prompting primary care consultation, particularly behavioural problems, self-harming behaviours and suicidal ideation, were especially important in predicting severe mental illness. Previous literature has stated the relevance of emotional dysregulation as a potentially important early sign of mental illness [11], and recognised EBD as an important target for prevention strategies [15]. This is particularly important considering that symptoms associated with higher levels of emotional dysregulation (e.g., self-harm) appear to be the stronger risk factors for later severe mental illness. In fact, there is a growing interest in considering emotional dysregulation the lowest common denominator of mental health problems [34].

### Clinical, research and policy implications

Our study suggests that primary care plays an important role in identifying patients at greater risk of later severe mental illness and that there are potentially identifiable symptoms that result in GP consultations in late childhood and early adolescence that are associated with higher severity and risk in mental health outcomes in young adulthood. Options such as routine monitoring for emotional or behavioural distress when addressing medically unexplained symptoms or in routine visits for other chronic problems could be considered, as well as follow-ups or appropriate referral pathways to other services when certain symptoms are detected. For example, screening for and appropriate attention to self-harming behaviours and suicidal ideation seem essential due to their significant association with increase in severe mental illness in early adulthood. It is vital that children and young people are signposted to early help, for example through schools and to evidence based digital interventions and, if at high risk of severe mental disorder, to specialist mental health services.

Early identification of symptoms that appear to be associated with later severe illness may be a potential route to reduce costs in the future in healthcare utilisation. Previous studies have found that EBD problems in childhood predict later prescriptions for psychotropic medications [35]. Given that most patients with mild to moderate mental disorders are managed in primary care, or referred for low intensity psychological interventions, referral to mental health services would generally be reserved for those with more severe illness [36].

Our findings suggest that policy directives focussed on early recognition of mental disorders in schools should extend to include primary care as a setting where early signs of severe mental health problems could be detected and early intervention initiated. Defining appropriate pathways to address these difficulties is key and an increasing area of need due to the rise of mental health difficulties in young people. Further training on identifying risk to self (e.g., self-harm, suicidal ideation) is key for prevention of further deterioration.

### Strengths and limitations

This study has significant strengths. The large sample size of primary care data allowed study of a large number of patients comprising a diverse population across several practices in the UK and contributed towards developing the identification of risk factors for identifying children and adolescents at risk of later severe mental illness, in order to support GPs. This study can also contribute to support future studies to identify what potential markers have strong associations with later severe mental illness and may require further study in order to develop prediction models that support treatment decisions and contribute to policymakers’ decisions. Our study sheds some light on potentially identifiable symptoms that predict higher mental illness severity and provides evidence to prioritise those that seem to be associated with higher levels of risk.

Several limitations should be noted. We have to consider the potential misclassification bias, changes in accuracy and completeness and reliability of clinical coding. We tried to minimise this effect with the hierarchical regression by controlling by practice. Furthermore, limited access to deprivation level or ethnicity data limited the extent of our ability to control for these variables.

Selection bias also has to be considered. Firstly, we selected our population on the basis of mental illness in young adults as recorded in primary care. This limits generalisation to those whose mental illness was not recorded in primary care, for multiple possible reasons. For example, our sample would not include participants diagnosed at 16 or 17 years who did not continue follow-up in primary care or moved somewhere else outside of the catchment areas. In addition, we are aware of the information loss when recoding variables into binary variables (e.g., absence/presence of symptoms) or categories (e.g. severe mental illness). Our study aimed to be exploratory in nature which suggests that further studies with greater specificity could be conducted using similar methodology. Further sources of potential confounding factors such as parental health seeking behaviour, parental mental illness or adverse environment conditions could not be accounted for.

### Future research

Future studies could shed more light on how specific symptoms are more relevant than others in predicting specific disorders. The use of a control group of young people who did not develop mental illness in young adulthood would also be a recommended future direction. The use of other outcomes associated with higher severity (e.g., inpatient admissions, intensive community treatment) should also be considered for future studies as a marker of severity.

Another potential area for further study is the predictive value of neurodevelopmental conditions, such as Attention Deficit Hyperactivity Disorder and Autism Spectrum Disorder, which has improved significantly over the last few years and was more likely to be unreliable when using a primary care database of decades of duration.

## Conclusions

Our study found that GPs records for EBD symptoms can be used to identify potential early risk markers for later mental illness. Self-harm, suicidal ideation, emotional and behavioural symptoms seem to be associated with a higher likelihood of later severe mental illness. Anxiety, sleep difficulties, eating symptoms and hyperactive behaviours in childhood were not significantly associated with severe mental illness in young adults. This study suggests that primary care data could be used to develop risk prediction models for identifying children and adolescents at risk of severe mental illness, in order to support primary care practitioners to detect and intervene early to help reduce adverse outcomes. These results can also support the development of referral pathways to the appropriate services (e.g., early intervention when presentations for self-harm).

## Supplementary Information

Supplementary Material 1.

## Data Availability

Data will not be available to the public as it requires specific permission to access CPRD data. However, for any researchers interested on accessing CPRD data, please see below link: https://www.cprd.com/data-access.
