# Supplementary material for: Emotional and behavioural symptoms in childhood (aged 8–14) and severity of mental illness outcomes in young adults: a retrospective observational study using cross-sectional data from electronic health records in the clinical practice research datalink (CPRD)
Source: BMC Pediatr. 2026 Jan 16;26:257. doi: 10.1186/s12887-025-06425-7 (PMC13045153; doi:10.1186/s12887-025-06425-7)
Supplement: Supplementary file 1 — Supplementary Material 1. [file 12887_2025_6425_MOESM1_ESM.docx]

**Appendices**

**Appendix A. Read codes**

**Appendix A.1. Read codes for emotional and behavioural dysregulation symptoms**

1. **Anxiety symptoms**

Eu41000 – [X]Panic disorder [episodic paroxysmal anxiety]

Eu93100 – [X]Phobic anxiety disorder of childhood

Eu40.00 – [X]Phobic anxiety disorders

Eu93000 – [X]Separation anxiety disorder of childhood

Eu93200 – [X]Social anxiety disorder of childhood

E200z00 – Anxiety state NOS

E200000 – Anxiety state unspecified

E200.00 – Anxiety states

E200300 – Anxiety with depression

1B13.00 – Anxiousness

E292400 – Adjustment reaction with anxious mood

E2D0100 – Childhood and adolescent fearfulness disturbance

E2D0000 – Childhood and adolescent overanxiousness disturbance

E2D0z00 – Disturbance anxiety and fearfulness childhood/adolescent NOS

E200200 – Generalised anxiety disorder

E200500 – Recurrent anxiety

Eu41.00 – [X]Other anxiety disorders

1. **Mood symptoms**

Eu32z14 – [X] Reactive depression NOS

8CAa.00 – Patient given advice about management of depression

E2Dy.00 – Other childhood and adolescent emotional problems

E113700 – Recurrent depression

Eu93z00 – [X]Childhood emotional disorder, unspecified

Eu32.00 – [X]Depressive episode

Eu32z00 – [X]Depressive episode, unspecified

Eu32100 – [X]Moderate depressive episode

Eu32400 – [X]Mild depression

Eu41200 – [X]Mixed anxiety and depressive disorder

E2D..11 – Adolescent - emotional problem

1B1U.00 – Symptoms of depression

E2D..00 – Disturbance of emotion specific to childhood and adolescence

8O82.00 – Emotional and psychosocial support and advice

1B1J.00 – Emotional problem

1B1J.11 – Emotional upset

1B17.00 – Depressed

1BT..00 – Depressed mood

6891.00 – Depression screen

1B17.11 – C/O - feeling depressed

E2Dz.00 – Childhood and adolescent emotion disorder NOS

1B1U.11 – Depressive symptoms

1. **Behavioural problems**

ZV40.11 – [V]Behavioural problems

Eu9..00 – [X]Behavioural/emotional disords onset childhood/adolescence

67IV.00 – Advice to carer regarding child's behaviour

1P5..00 – Aggressive behaviour

Eu92.00 – [X]Mixed disorders of conduct and emotions

E284.00 – Stress reaction causing mixed disturbance of emotion/conduct

3AB3.00 – Change in behaviour

E2C..11 – Behaviour disorder

1B1X.00 – Behavioural problem

13Z4C00 – Behavioural problems at school

E2C4z00 – Mixed disturbance of conduct and emotion NOS

1. **Hyperactivity symptoms**

1P00.00 – Hyperactive behaviour

1. **Eating symptoms**

ZC16.00 – Abuse of drugs to lose weight

ZC1..00 – Actions to lose weight

1613.00 – Appetite increased

R030000 – [D]Appetite loss

1615.00 – Reduced appetite

ZC15.00 – Self-induced vomiting to lose weight

1. **Sleep symptoms**

R005.00 – [D]Sleep disturbances

1BX1.00 – Excessive sleep

Eu51z11 – [X]Emotional sleep disorder NOS

1B1B.00 – Cannot sleep – insomnia

E274700 – Somnambulism - sleep walking

1. **Suicidal ideation**

1BD1.00 – Suicidal ideation

1BD3.00 - Suicidal plans

1. **Self-harm**

U2...00 – [X]Intentional self-harm

ZX...00 – Self-harm

ZX1..13 – Deliberate self-harm

ZX1..00 – Self-injurious behaviour

1. **Somatic symptoms**

R090N00 – [D]Nonspecific abdominal pain

R090E00 – [D]Recurrent acute abdominal pain

1B1G.00 – Headache

Eu50500 – [X]Vomiting associated with other psychological disturbances

1969.00 – Abdominal pain

E278100 – Tension headache

**Appendix A.2. Read codes for mental disorder diagnosis**

**A.2.1. Common mental illness**

**-Depressive disorders**

| Eu32z14 | [X] Reactive depression NOS |
| --- | --- |
| Eu32z11 | [X]Depression NOS |
| Eu32z12 | [X]Depressive disorder NOS |
| Eu32.00 | [X]Depressive episode |
| Eu32z00 | [X]Depressive episode, unspecified |
| Eu41211 | [X]Mild anxiety depression |
| Eu32400 | [X]Mild depression |
| Eu32000 | [X]Mild depressive episode |
| Eu41200 | [X]Mixed anxiety and depressive disorder |
| Eu32100 | [X]Moderate depressive episode |
| Eu32200 | [X]Severe depressive episode without psychotic symptoms |
| Eu32.11 | [X]Single episode of depressive reaction |
| E135.00 | Agitated depression |
| E2B..00 | Depressive disorder NEC |

**-Anxiety disorders**

| Eu40000 | [X]Agoraphobia |
| --- | --- |
| Eu41z00 | [X]Anxiety disorder, unspecified |
| Eu41100 | [X]Generalized anxiety disorder |
| Eu42.00 | [X]Obsessive - compulsive disorder |
| Eu41.00 | [X]Other anxiety disorders |
| Eu41000 | [X]Panic disorder [episodic paroxysmal anxiety] |
| Eu40.00 | [X]Phobic anxiety disorders |
| E202100 | Agoraphobia with panic attacks |
| E200300 | Anxiety with depression |
| E200200 | Generalised anxiety disorder |
| E203.00 | Obsessive-compulsive disorders |

**A.2.2. Severe mental illness**

**-Psychotic disorders**

| Eu20000 | [X]Paranoid schizophrenia |
| --- | --- |
| Eu25z11 | [X]Schizoaffective psychosis NOS |
| Eu20.00 | [X]Schizophrenia |
| Eu0z.12 | [X]Symptomatic psychosis NOS |
| Eu2z.00 | [X]Unspecified nonorganic psychosis |
| E103.00 | Paranoid schizophrenia |
| E107.00 | Schizo-affective schizophrenia |
| E10z.00 | Schizophrenia NOS |
| E10..00 | Schizophrenic disorders |
| E11..12 | Depressive psychoses |
| Eu32300 | [X]Severe depressive episode with psychotic symptoms |

**-Bipolar disorders**

| Eu31.11 | [X]Manic-depressive illness |
| --- | --- |
| Eu31.12 | [X]Manic-depressive psychosis |

**-Personality disorders**

| Eu60212 | [X]Antisocial personality disorder |
| --- | --- |
| Eu60200 | [X]Dissocial personality disorder |
| Eu60300 | [X]Emotionally unstable personality disorder |
| E21y200 | Borderline personality disorder |
| E215200 | Emotionally unstable personality |
| E21z.00 | Personality disorder NOS |
| E21..00 | Personality disorders |
| E210.00 | Paranoid personality disorder |
| Eu60513 | [X]Obsessive-compulsive personality disorder |

**-Eating disorders**

| Eu50000 | [X]Anorexia nervosa |
| --- | --- |
| Eu50200 | [X]Bulimia nervosa |
| Eu50z00 | [X]Eating disorder, unspecified |
| Eu50.00 | [X]Eating disorders |
| E271.00 | Anorexia nervosa |
| 1FF..00 | Binge eating |
| E275100 | Bulimia (non-organic overeating) |
| 1467.00 | H/O: anorexia nervosa |

**Appendix A.3. Comorbidities**

| **readcode** | **readterm** |
| --- | --- |
| E140.12 | Autism |
| Eu84500 | [X]Asperger's syndrome |
| Eu86.00 | [X]Neurodevelopmental delay |
| Eu84z11 | [X]Autistic spectrum disorder |
| Eu84011 | [X]Autistic disorder |
| F23..00 | Congenital cerebral palsy |
| 918e.00 | On learning disability register |
| Eu81z11 | [X]Learning disability NOS |
| F255011 | Focal epilepsy |
| F251500 | Tonic-clonic epilepsy |
| H33..11 | Bronchial asthma |
| H330.12 | Childhood asthma |
| C370.00 | Cystic fibrosis |
| C10..00 | Diabetes mellitus |
| 14G..00 | H/O: musculoskeletal disease |
| P63..00 | Congenital aortic valve stenosis |
| P6z..00 | Congenital heart anomaly NOS |
| F132300 | Myoclonic jerks |
| F251300 | Epileptic seizures - myoclonic |
| F132z12 | Myoclonic seizure |
| 2828.00 | Absence seizure |
| F250011 | Epileptic absences |
| B651.00 | Chronic myeloid leukaemia |
| J40..11 | Crohn's disease |
| J410100 | Ulcerative colitis |
| 43C3.11 | HIV positive |
| B641.00 | Chronic lymphoid leukaemia |
| N040.00 | Rheumatoid arthritis |
| N06z.11 | Arthritis |
| P6...00 | Other congenital heart anomalies |
| P68..00 | Congenital heart disease |
| K05..00 | Chronic renal failure |
| B681.00 | Chronic leukaemia NOS |
| C10E.00 | Type 1 diabetes mellitus |
| C109.12 | Type 2 diabetes mellitus |
| J101700 | Eosinophilic oesophagitis |
| C04..13 | Hypothyroidism |
| C02..11 | Hyperthyroidism |
| PJ0..00 | Down's syndrome - trisomy 21 |
| C380.00 | Obesity |
| C380300 | Morbid obesity |

**Appendix B. Age GP visit**

**Appendix B.1. Age of first GP visit for any reason at the age of 8-14**

|  | **Age** | **n** | **%** |
| --- | --- | --- | --- |
| **Age of first visit for any reason to GP** | 8 | 97,476 | 44,39 |
|  | 9 | 36,991 | 16,85 |
|  | 10 | 25,148 | 11,45 |
|  | 11 | 19,321 | 8,80 |
|  | 12 | 62,169 | 28,31 |
|  | 13 | 40,190 | 18,30 |
|  | 14 | 18,609 | 8,47 |

**Appendix B.2. Age of first GP visit for a mental disorder diagnosis at the age of 18-24**

|  | **Age** | **n** | **%** |
| --- | --- | --- | --- |
| **Age of first visit for a mental disorder diagnosis** | 18 | 27,613 | 17.08 |
|  | 19 | 27,552 | 17.04 |
|  | 20 | 26,230 | 16.23 |
|  | 21 | 23,685 | 14.65 |
|  | 22 | 21,362 | 13.21 |
|  | 23 | 18,871 | 11.67 |
|  | 24 | 16,341 | 10.11 |
